# Autoformer-Based Model for Predicting and Assessing Wheat Quality Changes of Pesticide Residues during Storage

**DOI:** 10.3390/foods12091833

**Published:** 2023-04-28

**Authors:** Yingjie Liu, Qingchuan Zhang, Wei Dong, Zihan Li, Tianqi Liu, Wei Wei, Min Zuo

**Affiliations:** 1 National Engineering Research Centre for Agri-Product Quality Traceability, Beijing Technology and Business University, Beijing 100048, China; 2 China Food Flavor and Nutrition Health Innovation Center, Beijing Technology and Business University, Beijing 100048, China; 3 School of Modern Post, Beijing University of Posts and Telecommunications, Beijing 100876, China

**Keywords:** wheat, wheat storage, quality assessment, prediction, Autoformer

## Abstract

Proper grain storage plays a critical role in maintaining food quality. Among a variety of grains, wheat has emerged as one of the most important grain reserves globally due to its short growing period, high yield, and storage resistance. To improve the quality assessment of wheat during storage, this study collected and analyzed monitoring data from more than 20 regions in China, including information on storage environmental parameters and changes in wheat pesticide residue concentrations. Based on these factors, an Autoformer-based model was developed to predict the changes in wheat pesticide residue concentrations during storage. A comprehensive wheat quality assessment index *Q* was set for the predicted and true values of pesticide residue concentrations, then combined with the K-means++ algorithm to assess the quality of wheat during storage. The results of the study demonstrate that the Autoformer model achieved the optimal prediction results and the smallest error values. The mean absolute error (MAE) and the other four error values are 0.11017, 0.01358, 0.04681, 0.11654, and 0.13005. The findings offer technical assistance and a scientific foundation for enhancing the quality of stored wheat.

## 1. Introduction

With an annual global yield of over 700 million tonnes, wheat is the primary grain crop grown in temperate regions [1], and one of the main reserve grains in China. Pesticides play an important role in preventing and reducing pest damage to grains, increasing their yield, and ensuring the quality of food crops [2]. Based on the most recent information available on the National Bureau of Statistics website, the annual production of wheat in China has reached 136.94 million tons. Along with the increasing production of wheat, quality problems during wheat storage have become increasingly prominent. As the primary source of energy for the majority of the world’s population, good-quality wheat is an important guarantee of food security and must be stored through an appropriate storage management system [3]. Pesticide residues are a significant factor that impacts the quality of wheat food. The increased use of pesticides leads to excessive accumulation of pesticide residues in wheat, and unlike other grains, pesticide residues cannot be eliminated through washing from the wheat kernel to the process of making wheat flour for direct consumption [4]. Therefore, controlling pesticide residue hazards during wheat storage is of paramount importance for improving the quality of wheat.

Pesticides such as dimethoate [5] and chlorpyrifos [6] are commonly used in agriculture to protect fruits, vegetables, and grains from pests and diseases during planting. Chlorpyrifos-methyl [7] is applied during storage to prevent losses from pest invasion. However, the widespread use of these pesticides leads to varying levels of pesticide residues in wheat crops after harvest [8], and cereal products can have lasting and comprehensive effects on public healthcare due to the presence of pesticide residues [9], potentially leading to chronic health problems such as damage to the neurological and immune systems with long-term exposure. Even after harvest, wheat grains may still contain pesticide residues, especially during storage. As storage time increases, wheat undergoes metabolic aging that can negatively affect its taste and color. Moreover, environmental factors during storage such as temperature [10] and humidity [11], as reasonably set environmental parameters, can accelerate the degradation of remaining pesticide residues in its seeds, ultimately affecting the quality of stored wheat grains. Therefore, it is essential to recognize the risks associated with pesticide residues in food and to take measures to minimize their impact on human health.

Faced with such a status quo, there is an increasing demand from the public for higher quality and quantity of cereal grains [12], resulting in a growing interest among scholars in the quality of wheat and other grains during storage. In past studies, Rakić et al. [13] investigated and analyzed the chemical properties of different varieties of wheat over time under predetermined storage conditions. Fazel-Niari et al. [14] classified different combinations of wheat seeds and other grains based on quality features such as shape, texture, and color using multiple classification methods based on a machine vision system of their own design, all with an accuracy of more than 90%. Kibar et al. [15] studied the changes in quality characteristics of two types of wheat under different storage environments for 6 months and found that unsuitable storage environments can be detrimental to the quality of wheat, eventually concluding that temperature is the environmental factor that most affects the quality of wheat. Yewle et al. [16] experimentally evaluated how germination rates and other qualities of grains with different moisture contents change under temporary sealed storage conditions to obtain the best storage period with the least impact on cereal quality. Liang et al. [17], based on low-field nuclear magnetic resonance imaging (LF-NMR) and differential scanning calorimetry (DSC) techniques, revealed complex multiple correlations among physical and chemical indicators of wheat. Nyarko et al. [18] used a new polypropylene bag to improve storage of grains, resulting in lower aflatoxin and pesticide residue concentrations, which in turn improved the quality of the grain seeds during storage. Escalante-Aburto et al. [19] used principal component analysis to distinguish the negative impact of different concentrations of moisture content on grain quality by assessing the biophysical and viscoelasticity of several types of grains, and finally analyzed the associations between quality characteristic variables using Pearson’s correlation analysis. In short, these studies have revealed various factors affecting grain quality and storage. Further research in this area is necessary to ensure sustainable agricultural practices and secure food supplies for our growing population.

Recently, time-series analysis [20] has been widely used in the fields of medicine [21,22], risk prediction [23,24], and agriculture [25,26], which is attributed to its ability to model and analyze historical information to predict upcoming tendencies The quality variation of the wheat storage process is closely related to the influencing factors with time-series characteristics, so there are more and more scholars of time-series analysis to predict and evaluate the quality of wheat storage and its related aspects. Jeong et al. [27] employed the random forest (RF) algorithm, which integrated four climatic variables and seven additional biophysical variables, to accurately forecast wheat yields across various regions worldwide. Agarwal et al. [28] made the assumption that the quality of stored wheat grains varies with time and extracted quality characteristics from wheat grain samples. Based on a support vector machine (SVM) classifier, they developed an automatic quality grading system for wheat during storage. With the application of deep learning methods, simple machine learning methods have the restriction of gradient explosion in high-dimensional data [29], so numerous researchers have directed their efforts to more complex neural network models. For example, Sindwani et al. [30] used recurrent neural networks (RNN) combined with factors such as CO_2_ concentration and the number of harmful insects in grain storage to predict the grain quality conditions in Indian grain silos. Duan et al. [31] and Yang et al. [32] used the long short-term memory neural network (LSTM) model to predict temperature and moisture, respectively, as elements which impact the quality of stored grain, providing a basis for setting better environmental parameters to safeguard the quality of stored grain. Jubair et al. [33] used an improved Transformer genomic prediction model to predict the fungal toxin content of barley. These studies demonstrate the potential of time-series analysis and machine learning methods for predicting and evaluating the quality of stored grain.

After a comprehensive review of the existing literature, this study presents a novel proposal that the concentration of pesticide residues in wheat storage is influenced by temperature and humidity during storage, which further affects the quality of wheat, so we assess the quality of wheat at different time points by evaluating the concentration of pesticide residues in wheat during storage. In order to complete this work, we first constructed an Autoformer-based model to predict pesticide residue changes in wheat during storage. Then a K-means++-based model was constructed for the quality assessment grading of wheat during storage. Between the two models mentioned above, the Autoformer model has advantages over other prediction models because the degradation of pesticide residues in wheat is a long process and the Autoformer model is also more suitable for dealing with long time-series data. In comparison, K-means++ has better classification results and algorithmic stability. By conducting comparative experiments, we found that the model in this study outperforms other similar models. The results have an important reference value for helping the technical managers of grain storage to adjust the settings of optimal storage conditions for wheat grain storage according to time points, promote the reduction rate of pesticide residues in wheat, and reduce the quality loss of stored wheat.

## 2. Materials and Methods

### 2.1. Materials

#### 2.1.1. Data

This study sourced its wheat storage monitoring data from five primary wheat-producing regions in China (East China wheat region, Central China wheat region, North China wheat region, Northwest China wheat region, and Southwest China wheat region), covering several provinces and more than twenty regions. Wheat was stored in 10 shallow round silos. We collected samples every day during the storage period, which were obtained by dividing the cuttings into three layers and subdividing each layer into a total of 13 sampling points. After mixing the samples from the entire bin, we obtained the final samples for analysis, which included a total of 2655 data points for the quality of wheat samples. In addition, the specific environmental parameters affecting the concentration of pesticide residues in wheat during storage in this study were storage temperature and humidity, and the experimental data involved storage humidities of 54%, 65%, and 75% and storage temperatures of 10 °C, 25 °C, and 35 °C. The Maximum Residue Limits of Pesticides in Food of the National Food Safety Standard [34] specifies the pesticide residue limits in wheat: 0.05 mg/kg for chlorpyrifos, 0.5 mg/kg for chlorpyrifos, and 5 mg/kg for chlorpyrifos-methyl. In the wheat storage monitoring dataset, most of the samples were below this limit. During the experimental process, as shown in Table 1, according to the ratio of 7:2:1, the experimental dataset was proportionally partitioned into three sets: training set, test set, and validation set.

#### 2.1.2. Experimental Environment

The experimental platform in this research utilized the PyTorch [35] deep learning framework, which is open-source software. The detailed parameters of the experimental environment are listed in Table 2.

### 2.2. Autoformer-Based Model for Predicting the Pesticide Residue Changes in Wheat

**Figure 1:**
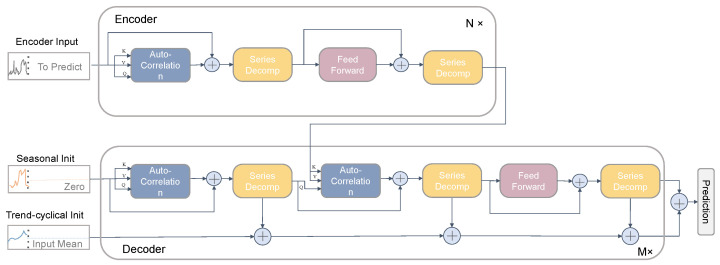
Autoformer model architecture.

In this paper, we construct a prediction model based on Autoformer [36] to predict changes in pesticide residues in wheat, which can realize the monitoring of pesticide residue concentration changes in wheat subjected to environmental factors during storage. Autoformer is an improved novel network model based on Transformer [37], with two main innovations: the introduction of decomposition architecture into the depth prediction model, and the design of a series-wise connected auto-correlation mechanism. These two innovative designs address two core problems inside the temporal prediction: complex temporal patterns and modeling the continuity of the temporal sequence itself [38]. The specific structure of Autoformer is shown in Figure 1.

The series decomposition block uses the traditional decomposition operation to flatten the period term and emphasize the trend term based on the sliding average idea for the pesticide residue data in wheat. The model alternately performs prediction result optimization and series decomposition, i.e., gradually separating trend and period terms from hidden variables to achieve progressive decomposition.

(1) $$\chi_{t}=Avgpool\left(Padding\left(\chi \right)\right)$$

(2) $$\chi_{s}=\chi -\chi_{t}$$

In the above equations, $\chi_{t}\in {\mathbb{R}}^{L\times d}$ stores the mean of each sliding window, i.e., the periodic fluctuations of the series, and $\chi_{s}\in {\mathbb{R}}^{L\times d}$ is the seasonal smoothed series retained after subtracting the short-term fluctuations, so the series decomp block can also be expressed as

(3) $$\chi_{s},\chi_{t}=SeriesDecomp\left(\chi \right)$$

Encoder: the input of Encoder will be the known raw time-series data. After this, the output result consists of two components, trend term and seasonal term, followed by gradual elimination of the trend term to obtain the period term $S_{en}^{l,1}$, $S_{en}^{l,2}$. Furthermore, based on this periodicity, an auto-correlation mechanism is used in Encoder to aggregate similar subprocesses of different periods to achieve the aggregation of information. The output of Encoder contains past seasonal information that will be used as crossover information to help the decoder optimize the prediction results. The whole process can be expressed as follows.

(4) $$S_{en}^{l,1},\_=SeriesDecomp(AutoCorrelation\left(\chi_{en}^{l-1}\right)+\chi_{en}^{l-1})$$

(5) $$S_{en}^{l,2},\_=SeriesDecomp(FeedForward\left(S_{en}^{l,1}\right)+S_{en}^{l,1})$$

(6) $$\chi_{en}^{l}=S_{en}^{l,2}$$

(7) $$\chi_{en}^{l,1}=EncoderBlk\left(\chi_{en}^{l-1}\right)$$

In the above equation, “_” denotes the trend part, which will not be used as the input to the decoder part, l∈{1,2,…,N} represents the lth encoder block, and $S_{en}^{l,i}$ denotes the i time-series decomposition lth encoder block.

Decoder: the Decoder input contains two parts, the trend part after sequence decomposition and other remaining parts. The Decoder part uses a two-way processing mode, a multi-stacked auto-correlation mechanism block for the seasonal part and a cumulative mechanism structure for the trend part accumulation, respectively. The upper branch processes the seasonal part, and the lower branch processes the trend-cyclical part. The upper branch first uses auto-correlation to extract the temporal dependencies inherent in the future predicted states, then uses encoder–decoder auto-correlation to extract information from the historical series with higher-order temporal dependencies from the encoder output, and finally passes through the Feed Forward layer. The lower branch gradually extracts the trend information from the predicted hidden variables, and the outputs of each sub-layer of the upper branch are summed together using the weighted addition method. The specific equation is shown as follows.

(8) $$S_{de}^{l,1},T_{de}^{l,1}=SeriesDecomp\left(AutoCorrelation\left(\chi_{de}^{l-1}\right)+\chi_{de}^{l-1}\right)$$

(9) $$S_{de}^{l,2},T_{de}^{l,2}=SeriesDecomp\left(AutoCorrelation\left(S_{de}^{l,1},\chi_{en}^{N}\right)+S_{de}^{l,1}\right)$$

(10) $$S_{de}^{l,3},T_{de}^{l,3}=SeriesDecomp\left(FeedForward\left(S_{de}^{l,2}\right)+S_{de}^{l,2}\right)$$

(11) $$T_{de}^{l}=T_{de}^{l-1}+W_{l,1}*T_{de}^{l,1}+W_{l,2}*T_{de}^{l,2}+W_{l,3}*T_{de}^{l,3}$$

(12) $$\chi_{de}^{l}=S_{de}^{l,3}$$

**Figure 2:**
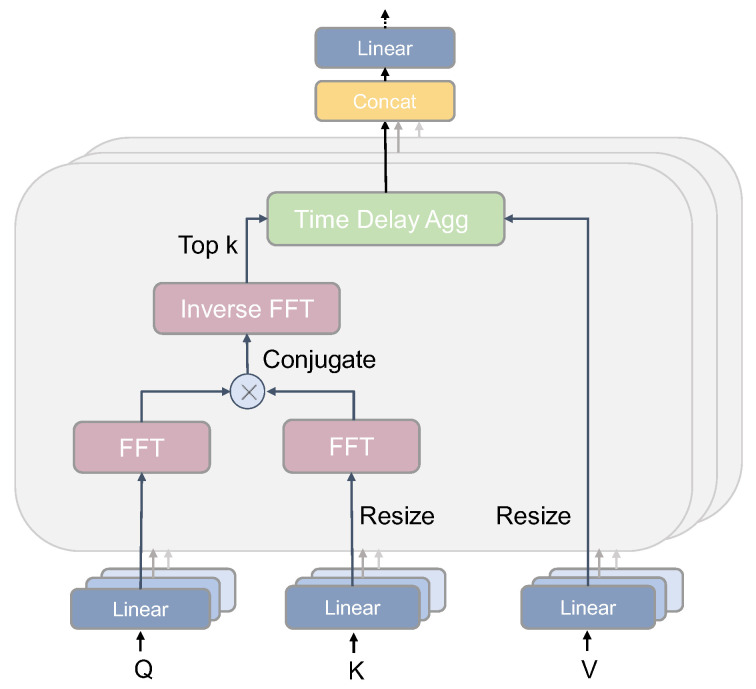
Auto-correlation architecture.

Autoformer’s core auto-correlation mechanism, which is key to discovering period-based dependencies by calculating sequence auto-correlation coefficients, consists of period-based dependencies and time-delay aggregation. Figure 2 shows a schematic diagram of the internal structure of the auto-correlation module.

The dependence of the period in the auto-correlation mechanism is found from the efficient computation of the auto-correlation coefficients, as shown in Figure 2, by mapping the inputs to obtain Q, K, and V. Based on the Winer–Khinchin theory [39], a Fast Fourier Transforms (FFT) operation is performed on Q and K, respectively, and a conjugate operation is also added to K. Finally, the original correlation coefficient ${ℛ}_{XX}(\tau )$ of the sequence and the lag sequence must be computed to find the periodically similar subsequence. The specific formula is as follows.

(13) $$S_{XX}(f)=ℱ(W^{Q}X_{t}){ℱ}^{*}(W^{K}X_{t})$$

(14) $${ℛ}_{XX}(\tau )={ℱ}^{-1}(S_{XX}(f))$$

In the above equation, ℱ represents the FFT and ${ℱ}^{-1}$ is the reciprocal of ℱ, ∗ represents the conjugate operation, and $S_{XX}(f)$ is in the frequency domain.

**Figure 3:**
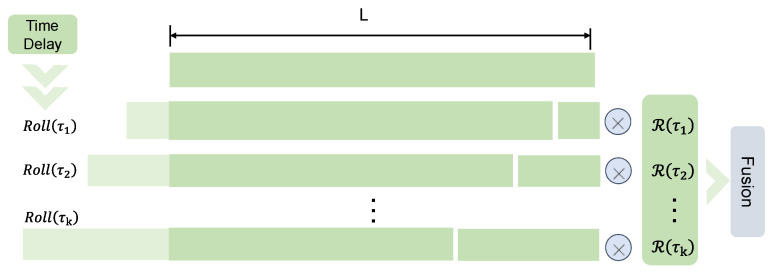
Time-delay aggregation.

Next, time-delay aggregation of auto-correlation first aligns the subsequences with the same phase based on the estimated cycle length using the Roll() operation, converts them into probabilities by the softmax normalization operation, and finally aggregates the information by fusion. The Roll() operation moves V into the same phase, as shown in Figure 3.

### 2.3. K-Means++-Based Model for Wheat Quality Assessment

In order to better assess the quality of stored wheat during storage, a comprehensive evaluation index *Q* was constructed in this study, which integrates the present and predicted pesticide residue concentrations of wheat; the equation of the evaluation index *Q* is shown in (15).

(15) $$Q=\left[d_{i},{\overset{-}{d}}_{i}\right]$$

In the formula, $d_{i}\;$∈ {1, 2, …, n} denotes the true value and $d_{i}\;$∈ {1, 2, …, n} denotes the mean of the predicted values in the next n days.

During the study, since the wheat quality data were small-sample data with little noise, the clustering method K-means++ [40] was used to assess the quality of wheat during storage. K-means++ is an optimization of the K-means random initialization cluster center method. Unlike K-means, the core idea of K-means++ is that when selecting a new cluster center, the more distant the point from the existing cluster center, the more likely it has the opportunity to be selected as the cluster hub. The K-means++ algorithm does not require artificial determination of the initial cluster center, but performs better on high-dimensional data, which is why it was used for the analysis of wheat quality data during the experiment.

**Figure 4:**
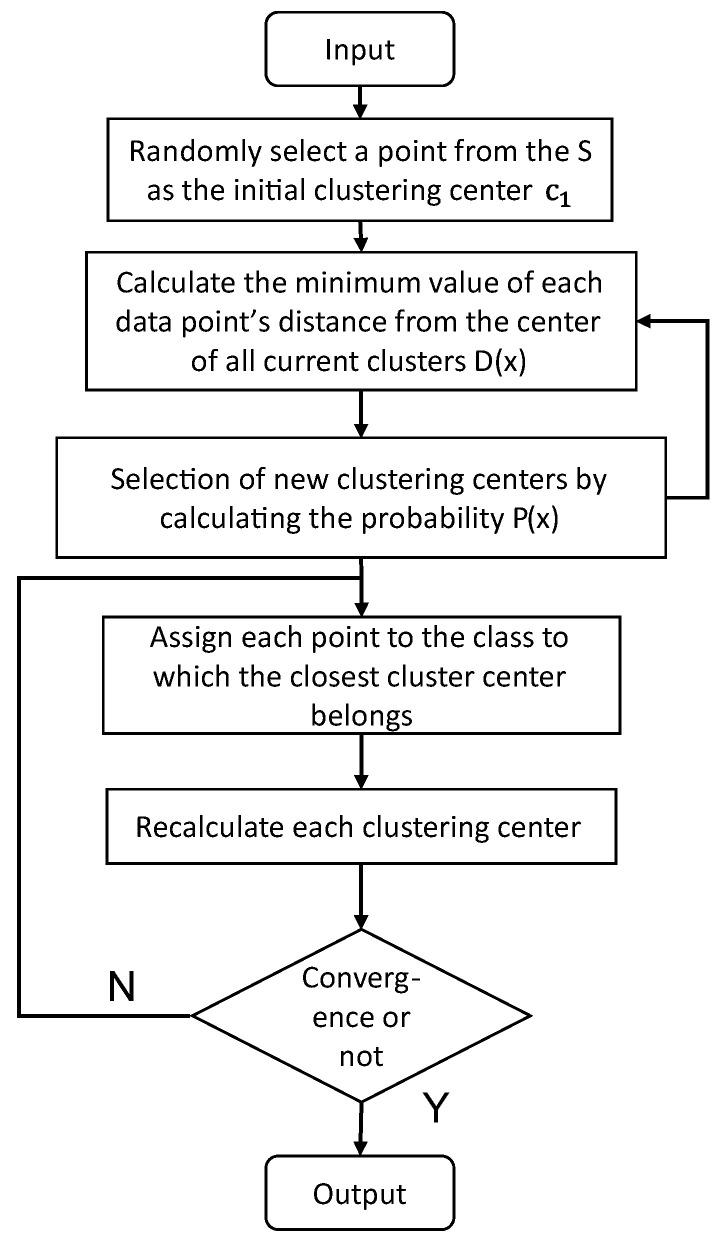
K-means++ algorithm flow chart.

Given dataset S with N data points, let k be the desired number of clusters. The algorithm outputs a set of k centroids {$\;c_{1}$, $c_{1}$, …, $c_{k}$} that minimize the within-cluster sum of squares. The standard algorithm form of the K-means++ algorithm is shown below. Furthermore, the detailed algorithm flow is shown in Figure 4.

(1) Randomly select a point from S as the initial clustering center $c_{1}$.
(2) For each data point x in S, calculate the minimum distance between each point and the currently existing clustering center, denoted by D(X).
(3) Select the next cluster center $c_{i}$ from S with probability $P\left(x\right)=\frac{D{\left(X\right)}^{2}}{\sum_{x\in X}D{\left(X\right)}^{2}}$ and add it to the set of selected clustering.
(4) Iterate step (2) and step (3) up to k clustering centers.
(5) Execute the standard K-means algorithm to allocate data points to the closest cluster centers and modify the locations of the cluster centers until convergence.

### 2.4. Model Evaluation Metrics

The evaluation of wheat quality relies on analyzing the changes in pesticide residue levels as wheat is exposed to different environmental conditions during storage. Hence, the ability to accurately predict changes in pesticide residues will affect the accuracy of wheat quality assessment.

#### 2.4.1. Evaluation Metrics for Predictive Models

Five assessment criteria, including MAE, mean squared error (MSE), mean absolute percentage error (MAPE), root-mean-square error (RMSE), and symmetric mean absolute percentage error (SMAPE), were employed in this study to gauge the fluctuations in pesticide residue levels in wheat over the course of storage. These measures were computed using the following formulae:

(16) $$MAE=\frac{1}{n}\overset{n}{\underset{i=1}{\sum }}|{\hat{d}}_{i}-d_{i}|$$

(17) $$MSE=\frac{1}{n}\overset{n}{\underset{i=1}{\sum }}{\left({\hat{d}}_{i}-d_{i}\right)}^{2}$$

(18) $$MAPE=\frac{100\%}{n}\overset{n}{\underset{i=1}{\sum }}|\frac{{\hat{d}}_{i}-d_{i}}{d_{i}}|$$

(19) $$RMSE=\sqrt{\frac{1}{n}\overset{n}{\underset{i=1}{\sum }}{\left({\hat{d}}_{i}-d_{i}\right)}^{2}}$$

(20) $$SMAPE=\frac{100\%}{n}\overset{n}{\underset{i=1}{\sum }}\frac{|{\hat{d}}_{i}-d_{i}|}{\left(|{\hat{d}}_{i}|-|d_{i}|\right)/2}$$

In the above formulae, ${\hat{d}}_{i},i\;$∈ {1, 2, …, n} represents the predicted value, $d_{i},i\;$∈ {1, 2, …, n} represents the true value, and n represents the quantity of indicator variables. The range of these evaluation indicators is [0, +∞), and the evaluation metrics reach their minimum value of 0 while the predicted value is identical to the true value, at which time the prediction model achieves the optimal effect for the ideal state of the model. When the error is larger, the value of the evaluation indicators is larger, which can be used as a reference.

#### 2.4.2. Evaluation Metrics for Clustering Models

For the clustering results acquired through the K-means++ algorithm, we used the silhouette coefficient (SC) [41] and the Davies–Bouldin index (DBI) [42]. The objective of using these indicators was to determine the ideal number of clusters for the classification of wheat quality during storage and achieve accurate classification.

The silhouette coefficient is calculated as follows:

(21) $$SC=\frac{1}{N}\overset{N}{\underset{i=1}{\sum }}\frac{b\left(i\right)-a\left(i\right)}{max\left\{a\left(i\right),b\left(i\right)\right\}}$$

In Equation (21), N represents the quantity of training samples, a(i) represents the average distance between sample points and other points within the same cluster, and b(i) denotes the mean distance between a sample point and all points in its nearest cluster. The SC takes values in the range of [−1, 1], with larger values indicating superior clustering performance.

The Davies–Bouldin Index is calculated as follows:

(22) $$DBI=\frac{1}{K}\overset{N}{\underset{1}{\sum }}\max\left(\frac{s_{i}+s_{j}}{d_{ij}}\right)$$

In Equation (22), s represents the mean distance between each point of the cluster and the center of mass of the cluster, also known as the cluster diameter, and $d_{ij}$ denotes the distance between its different clustering cluster centers i  and  j. The formula calculates the maximum similarity of a total of K clusters taking the mean value, and the best clustering is achieved when the DBI reaches a minimum value.

## 3. Results and Discussion

### 3.1. Dataset of Wheat Quality Assessment

**Figure 5:**
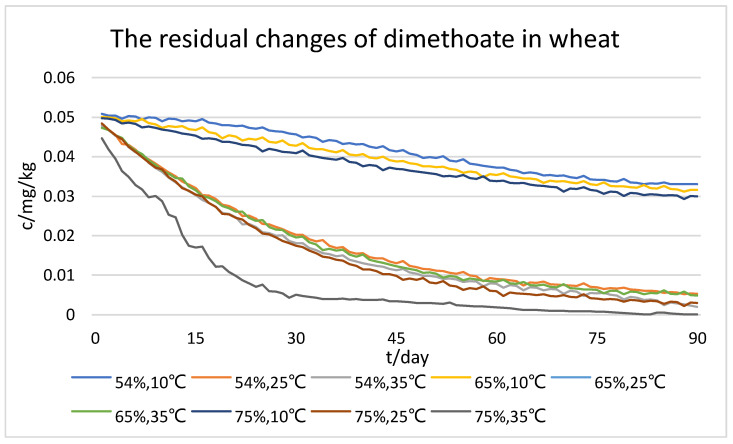
Dataset of residual changes of dimethoate in wheat.

**Figure 6:**
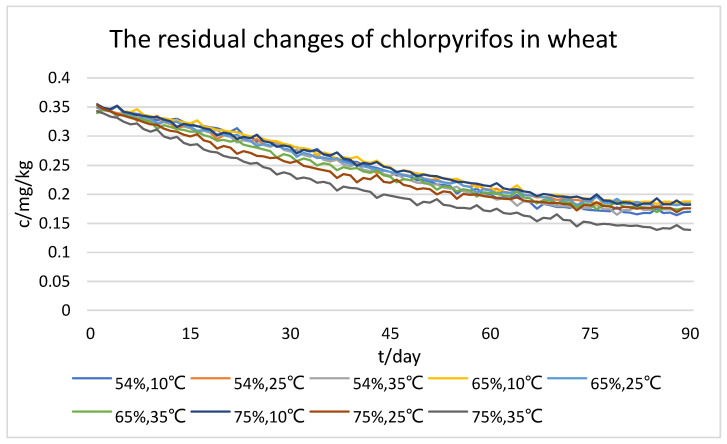
Dataset of residual changes of chlorpyrifos in wheat.

**Figure 7:**
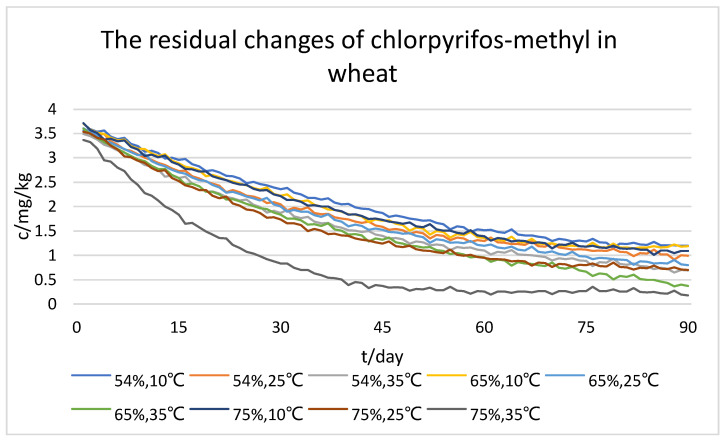
Dataset of residual changes of chlorpyrifos-methyl in wheat.

According to the characteristics of pesticide residue degradation in the process of grain storage, this study used a 90-day period to collect experimental data on six dimensions of information: time, temperature, humidity, and concentrations of dimethoate, chlorpyrifos, and chlorpyrifos-methyl. The concentrations of these pesticides were used as predictors, while moisture content and temperature were treated as control variables. Figure 5, Figure 6 and Figure 7 partially display the changes in pesticide residue levels during wheat storage.

As shown in the figures, we can initially see that all three types of pesticide residue in wheat gradually degraded with time. The higher the temperature, the faster the concentration of pesticide residues decreased under the same storage humidity; also, the higher the humidity, the faster the concentration of pesticide residues decreased under the same storage temperature. However, comprehensively comparing the three types of pesticide residue, the degradation rates of dimethoate and chlorpyrifos-methyl were the fastest and the most affected by temperature; humidity had a facilitating effect on the degradation rate of two pesticides, but the effect was not significant enough. In addition, the degradation rate of chlorpyrifos was less affected by both temperature and humidity. From the results of the dataset, it can be seen that the optimum storage humidity for pesticide residue degradation is 75%, and the optimum temperature is 35 °C.

### 3.2. Comparison of Models for Predicting Changes in Pesticide Residues in Wheat

To demonstrate the effectiveness of the prediction model in the research, the Autoformer model was compared with several existing widely used neural network prediction models. For the same wheat pesticide residue quality assessment dataset, RNN, LSTM, and Transformer were used in this study to conduct comparison experiments with Autoformer. Five model evaluation metrics, MAE, MSE, MAPE, RMSE, and SMAPE, were also employed to estimate the prediction capabilities. In Table 3, these results are presented in detail.

The Autoformer model achieved MAE, MSE, MAPE, RMSE, and SMAPE values of 0.11017, 0.01358, 0.04681, 0.11654, and 0.13005, respectively, and as can be seen from the previous section, the larger the value of the evaluation index of the prediction model, the larger the error of the prediction. It is also evident from the results in Table 3 that among the four compared prediction models, the difference between the error values of RNN and LSTM is not significant, but their error values are significantly higher than those of the latter two prediction models. In particular, the SMAPE values are as high as 7.32437 and 6.43174, respectively. On the other hand, the error values of the Transformer and Autoformer models are closer and smaller than those of the first two prediction models, indicating that these two models are able to predict more accurately the target variables. However, comparing the model evaluation metrics, the values of MAPE, RMSE, and SMAPE for the Transformer model were higher than those of the Autoformer by 0.23574, 0.08282, and 2.30708, respectively, indicating that the comprehensive performance of Transformer prediction is inferior to Autoformer. In a word, the Autoformer-based prediction model achieved the highest prediction accuracy on the stored wheat quality dataset, exhibiting superior performance and outperforming the remaining three prediction models.

### 3.3. Comparison of Clustering Models for Wheat Quality Assessment

**Figure 8:**
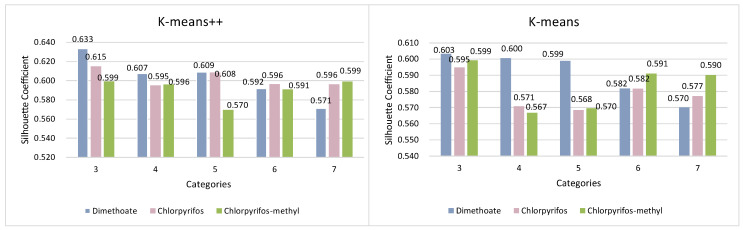
Comparison of Silhouette Coefficient of different clustering algorithms.

**Figure 9:**
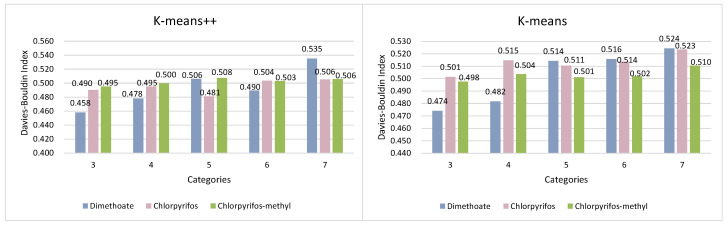
Comparison of Davies–Bouldin Index of different clustering algorithms.

According to the results of the prediction of pesticide residue changes based on Autoformer, for further work on wheat quality assessment, the daily evaluation index *Q* values of individual samples of wheat were used as the clustering feature input to the model. While applying the K-means++ and conventional K-means algorithms for wheat quality assessment of three pesticide residues in wheat storage grains, in order for the experiments to be more useful, two clustering evaluation indexes SC and DBI were used in this paper to evaluate their clustering results reasonably, while conducting experiments. The findings are presented in Figure 8 and Figure 9.

As shown in Figure 8, for different clustering algorithms, the SC of the wheat quality data decreased gradually as the number of clusters increased, and the SC of the two clustering algorithms reached a maximum evaluation index value when there were three clusters. The SC values were 0.633 and 0.603, respectively, when the wheat quality data were most dispersed among clusters and had the best clustering quality.

Figure 9 presents the results of the DBI applied to the wheat quality data with three to seven clusters. As shown in the figure, the magnitude of the DBI value shows a gradual increase in general, and unlike the SC clustering evaluation index, the DBI reaches its optimal classification result for clustering at the minimum value. Clearly, the best clustering effect of the two models is achieved when there are three clusters. At this time, the DBI values of the two clustering algorithms are 0.458 and 0.474, respectively.

**Figure 10:**
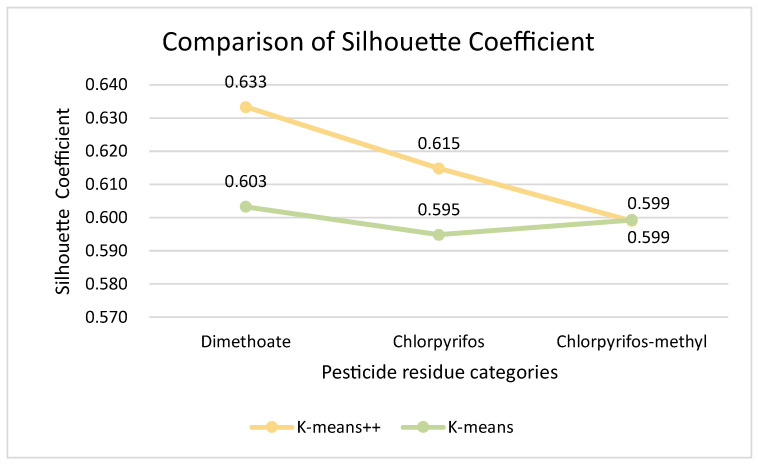
Comparison of Silhouette Coefficient.

**Figure 11:**
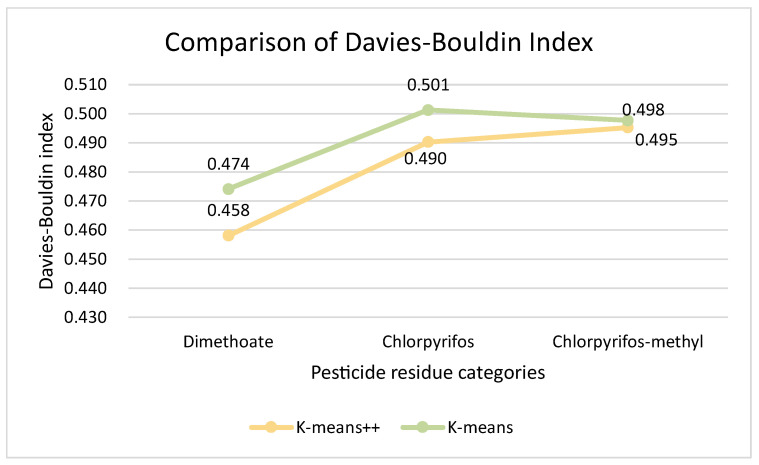
Comparison of Davies–Bouldin Index.

Comparing the evaluation indexes of the two clustering models together, we can easily see that for the wheat quality assessment dataset, despite the use of different clustering algorithms, both have the best results in terms of classification results when there are three clusters, i.e., classifying wheat quality into three levels. Figure 10 and Figure 11 present results of the comparison of the evaluation indexes of the two clustering algorithms when there are three clusters.

After comparing the evaluation metrics for both the SC and DBI with three clusters, according to the line graphs, we concluded that the K-means++ clustering model performed better than K-means for wheat quality assessment during storage. Therefore, K-means++ was used as the clustering model for wheat quality assessment during storage in this study, and in this way the wheat quality variation dataset was divided into three categories according to the K-means++ algorithm model. The wheat quality data of different categories corresponded to the corresponding quality levels, and the two-dimensional clustering centers of the three pesticide residue concentrations in wheat and the results of the number of samples in each class are shown in Table 4.

As shown in Table 4, the clustering results show that the quality of wheat became worse with an increase in the index value of the cluster center, which is also consistent with the reality of grain storage, the lower the concentration of pesticide residues in silos, the better the quality condition of wheat will be. As shown in Table 4, in terms of pesticide residues of dimethoate, most samples in the wheat quality dataset are at level 2: there are 1080 of these samples, accounting for about 41% of the total samples, and the clustering centers of the three different levels do not differ much. This is followed by the concentration of chlorpyrifos residues in the wheat quality data. Most samples belong to level 3: there are 1035 of these samples, accounting for about 39% of the total samples. However, compared to the other two types of pesticide residue, its sample data for the three types of quality level of the clustering center differences are small, which may be caused by the degradation of chlorpyrifos in the grain bin being less affected by the temperature and humidity. Finally, the residual levels of chlorpyrifos-methyl, on the other hand, were relatively evenly distributed among the quality data samples of wheat, but the values of their cluster centers were more varied, which indicated that the degradation of chlorpyrifos-methyl was the fastest among the quality data of wheat. In conclusion, the above results indicate that different types of pesticide residue are degraded at different rates during wheat storage under the influence of different storage environment parameters, thus affecting the quality of wheat.

Dimethoate, chlorpyrifos, and chlorpyrifos-methyl were used as the three most common pesticide residues in cereals to measure the quality assessment of wheat. The results suggested that all three pesticide residues degraded gradually over time, and the rate of degradation became faster with increasing storage temperature and humidity. Of these, temperature is the main element affecting the degradation of pesticide residues in wheat. Moreover, the concentration of chlorpyrifos in the results degraded slowly and was less dependent on the environmental conditions of storage. This may be due to a number of reasons, one being the properties of chlorpyrifos itself, such as chemical structure or volatility, the other is the influence of the type of grain, as the rate of degradation of the same pesticide residue in various grains may be different. Pesticide residues in the natural environment degrade faster due to factors such as sunlight, wind, and precipitation. However, in the process of grain storage, storage conditions are mostly airtight and protected from light. The chemical properties of pesticide residues during storage are more stable and not easily degraded, posing a threat to the quality of wheat. Furthermore, unlike other grains, wheat is generally stored for a longer period of time after harvest. In order to ensure quality during storage, for pesticides with slow degradation rates during storage and low national limit standards, such as chlorpyrifos, according to the degradation rate of such pesticides, to strictly limit its detection limit before storage. In addition, for chlorpyrifos-methyl, the grain protectant with a faster degradation rate and higher national limit standards, it is necessary to master its application cycle in the storage process.

During storage, there are a variety of elements that affect the quality of wheat, and it is scientific to assess wheat quality by changes in pesticide residue concentrations in wheat. Along with the high-speed growth of computer technology, deep-learning-related technology, which can automatically learn and improve the feature extraction of quality, has created new ideas for wheat quality detection and assessment. By monitoring and predicting the quality changes of wheat during storage, and assessing the quality condition of wheat in grain storage in a timely manner, grain storage monitors can be inspired to timely adjust the storage environment conditions to control the degradation of pesticide residues, enhancing the quality condition of wheat.

## 4. Conclusions

Playing an essential role in cereal crops, wheat is an indispensable food in people’s daily life, especially for food security in a country with a large population such as China, so its quality is very important. The use of pesticides in large quantities has increased the yield of wheat, but the safety of grain quality from pesticide residues has also attracted attention. These pesticide residues may affect the quality of wheat, and in serious cases may even be potentially harmful to human health.

The quality of wheat is mainly affected by the harvest time and storage conditions. In this study, starting from the storage environment, we used the temperature, storage humidity, and the concentration of pesticide residues in wheat during storage, which are elements that can easily contribute to the quality of wheat, and then innovatively combined the degradation changes of pesticide residues occurring during wheat storage. We used an Autoformer-based deep neural networks model to accurately predict the levels of different pesticide residues in wheat under different environmental influences in grain storage, and an evaluation index *Q* was constructed using existing and predicted pesticide residue concentrations to make a reasonable grading assessment of the quality of wheat. The results showed that the degradation speed of pesticide residues during storage is influenced by environmental parameters such as temperature and humidity, and considering the three pesticide residues together, the most suitable environmental parameters for their residue dissipation were a storage humidity of 75% and a temperature of 35 °C. Therefore, suitable storage conditions will promote the degradation of pesticide residues in wheat.

However, it should be noted that our study only investigated the effects of two major environmental factors on the degradation of pesticide residues during storage. Other factors such as the pH value of the storage environment and the moisture content of the wheat itself could also impact the degradation of pesticide residues in wheat storage and warrant further investigation. Additionally, beyond pesticide residues, other quality indicators such as cultivar, fungal and bacterial content, sensory features, degree of oxidation, etc., could also be considered in future quality assessments of stored wheat.

## Figures and Tables

**Table 1:** Partitioning of datasets in experiments.

Dataset	Training Set	Test Set	Validation Set
2655	1858	531	266

**Table 2:** Experimental platform and environmental parameters.

**Computer information**	Operating system	Windows 10 64-bit
	CPU	Intel(R) Core(TM) i5-8265U CPU @ 1.60 GHz (8 CPUs) ~1.8 GHz
	GPU	Radeon 540X Series
	RAM	16 GB
**Toolkit**	Python 3.7	Numpy 1.21.5
		Scikit_Learn 1.0.2
		Pandas 0.25.1
		Torch 1.12.0
		Matplotlib 3.5.2

**Table 3:** Experimental results on the error comparison of Autoformer-based wheat pesticide residue changes prediction models.

Model	MAE	MSE	RMSE	MAPE	SMAPE
RNN	0.28169	0.04516	0.88492	0.55132	7.32437
LSTM	0.24352	0.04324	0.83203	0.43147	6.43174
Transformer	0.16428	0.03986	0.28255	0.19966	2.43713
Autoformer	0.11017	0.01358	0.04681	0.11654	0.13005

**Table 4:** Clustering centers and ranking of the three clusters of wheat.

Categories	$d_{i}$	$d_{i}$	Sample Size	Quality Level
Dimethoate 1	0.03950035	0.03341637	810	Level 1
Dimethoate 2	0.04434314	0.03039072	1080	Level 2
Dimethoate 3	0.04900005	0.04175411	765	Level 3
Chlorpyrifos 1	0.22891236	0.17001536	855	Level 1
Chlorpyrifos 2	0.28989454	0.20340521	765	Level 2
Chlorpyrifos 3	0.33112902	0.25336484	1035	Level 3
Chlorpyrifos-methyl 1	1.77091609	1.22453220	890	Level 1
Chlorpyrifos-methyl 2	2.43346228	1.54720279	935	Level 2
Chlorpyrifos-methyl 3	3.20258731	2.02372957	830	Level 3

## Data Availability

The data used to support the findings of this study are available from the corresponding author upon request.
